# Optical sparse aperture imaging with faint objects using improved spatial modulation diversity

**DOI:** 10.1038/s41598-017-17844-7

**Published:** 2017-12-13

**Authors:** Zongliang Xie, Haotong Ma, Bo Qi, Ge Ren, Xiaojun He, Li Dong, Yufeng Tan, Shan Qiao

**Affiliations:** 1 Key Laboratory of Optical Engineering, Chinese Academy of Sciences, Chengdu, 610209 China; 2 Institute of Optics and Electronics, Chinese Academy of Sciences, Chengdu, 610209 China; 3 University of Chinese Academy of Sciences, Beijing, 100039 China; 4 College of Opto-Electronic Science and Engineering, National University of Defense Technology, Changsha, 410073 China

## Abstract

The next generation of optical sparse aperture systems will provide high angular resolution for astronomical observations. Spatial modulation diversity (SMD) is a newly developed post-processing technique for such telescopes, faced with challenges of imaging faint objects, which are very attractive for astronomers but always make raw diversity images suffer serious photon noise. In this paper, we propose an improved SMD with denoising reprocessing embedded to address the problem. The blocking-matching and 3D filtering algorithm, a state-of-the-art denoising technique, is first employed to process the diversity images with low photon intensities generated by spatial modulation, specifically switching off each sub-aperture sequentially. SMD algorithm then can be applied to estimate wavefront and digitally restore images. It is demonstrated by both simulations and experiments that the proposed method outperforms the previous SMD in terms of reconstructions of wavefront and imagery from the raw images of faint objects corrupted seriously by photon noise. The reported method may provide an alternative approach to acquire high-quality images of faint objects for astronomical observations of the future segmented mirrors or telescope arrays.

## Introduction

Due to the limits to the size and weight of monolithic primary mirrors, it’s difficult for modern ground-based and space-based telescopes to achieve the desired increases in angular resolution. Optical sparse aperture systems, capable of using multiple small apertures to realize the high resolution equivalent to a large single one, has grown out of the quest for the next generation of telescopes. Such a sparse aperture system mainly has two implementation forms: the segmented mirror and telescope array. Many segmented telescopes have been built or are under construction, such as the James Webb Space Telescope (JWST)^1^, Thirty Meter Telescope (TMT)^2^, and Extremely Large Telescope (ELT)^3^. There are also some telescope arrays being tested, such as Large Binocular Telescope (LBT)^4^, Adaptive Reconnaissance Golay-3 Optical Satellite (ARGOS)^5^, and Star-9^6^. Instead of practical projects, ARGOS and Star-9 are experimental testbeds. Installed in a simulated space environment, ARGOS aims to explore solutions for potential problems that may occur in space^5^. STAR-9 successfully combines nine sub-telescopes within a wide phased field^6^.

Spatial modulation diversity (SMD) is a newly developed post-processing method for such sparse aperture systems^7^, sharing its roots with phase diversity (PD)^8–10^, an image-based wavefront sensing and image restoration approach allowing aberrations to be estimated from diversity images generated by loading known phase, usually different amounts of defocus. Alternatively, considering the special configuration of sparse aperture systems, SMD produces diversity images by spatial modulation, specifically controlling the transmittance of sub-apertures. Then based on the diversity raw images, an optimization process is performed to recover the aberrations and then a high-quality image can be acquired with deconvolution. It may provide an alternative solution for adaptive optics. Compared with traditional PD, SMD eliminates the needs of multiple image planes, defocus optics or high precision actuators, at the expense of taking several snapshots over time, compacting the system and reducing influences of actuator’s precision. Prior work has also demonstrated SMD’s superior reconstructions^7^, and its availability for a real binocular telescope testbed^11^.

Despite these advantages and demonstrations of SMD, there is still a challenge of imaging faint astronomical objects. To guarantee the object of interest being in the same isoplanatic patch during the total time of multi-snapshots, the spatial modulation and the corresponding exposures should be performed within a short time, the maximum of which might be 0.02 seconds as indicated in image-based adaptive optics^12^. There are many modulation devices well beyond the requirements, such as spatial light modulators and digital micromirror devices with response speeds of kHz rate. The problem of imaging faint objects is that the raw images captured within such a short time will suffer from serious Poisson noise due to the photon detection, thereby with essential details submerged into the noises. Inevitably, the lost image information will decreases the accuracy of wavefront reconstruction and influences the quality of image reconstruction^13^.

In this paper, we propose an improved SMD (ISMD) for faint astronomical objects, adopting a helpful strategy which is embedding a denoising preprocessing step into the previous technique. Diversity images of the faint objects at low photon levels are yielded by switching off the sub-apertures in a certain order. Then a state-of-the-art denoising technique, specifically the blocking-matching and 3D filtering (BM3D) algorithm^14,15^, is used to preprocess the noisy data set. Such an algorithm can produce images equivalent to those corresponding to bright objects with high signal-to-noise ratios. The previous SMD algorithm can be then applied to estimate the aberrations and computationally reconstruct images. In SMD algorithm, we only need to guarantee the diversity image and OTF generated by the same spatial modulation corresponding to each other regardless of the modulation order. Simulation results show that the proposed ISMD can achieve higher accuracy of wavefront reconstruction and better quality of recovered images compared with previous SMD. It is further presented by experimental study that the wavefront and image reconstructions using ISMD at high noise levels exhibit the similar quality to those using SMD at high photon levels. By proposing such a useful strategy, SMD is developed to be capable of imaging faint objects, which are very attractive for astronomy. It is believable that the proposed ISMD may find wide applications in post processing of astronomical observations of the future segmented mirrors or telescope arrays.

## Results

### Quantitative metric

To quantitatively evaluate the results, we introduce some metrics. The peak signal-to-noise ratio (PSNR) is utilized to denote the noise level, expressed as

1 $${\rm{PSNR}}=10\,\mathrm{log}\,10(\frac{max{(o(x))}^{2}}{{\sum }_{x}{(o(x)-d(x))}^{2}/N}),$$

where $o(x)$ is the ideal image without noise, $d(x)$ is the noisy image and *N* is the number of total pixels, and $x$ is a 2D vector in image plane. The root-mean-square (RMS) is used here to evaluate the accuracy of aberration estimations, which is defined as

2 $${\rm{RMS}}=\sqrt{\sum _{u}{({\phi }_{r}(u)-\phi (u))}^{2}/{N}_{pupil}},$$

where ${\phi }_{r}(u)$ is the recovered wavefront, $\phi (u)$ is the loaded wavefront, and ${N}_{{pupil}}$ is the number of total pixels in the sampled pupil and *u* is a 2D vector in pupil plane. The correlation coefficient (Co) is selected to measure the quality of image reconstruction, defined below

3 $${\rm{Co}}({\rm{A}},{\rm{B}})=cov(A,B)/{\sigma }_{A}{\sigma }_{B},$$

where ${cov}(A,B)$ is the cross-covariance between the ideal image *A* and the recovery *B*, and ${\rm{\sigma }}$ denotes the standard deviation. Ranging in zero and one, bigger Co values indicate better reconstructions.

### Numerical simulation

**Figure 1:**
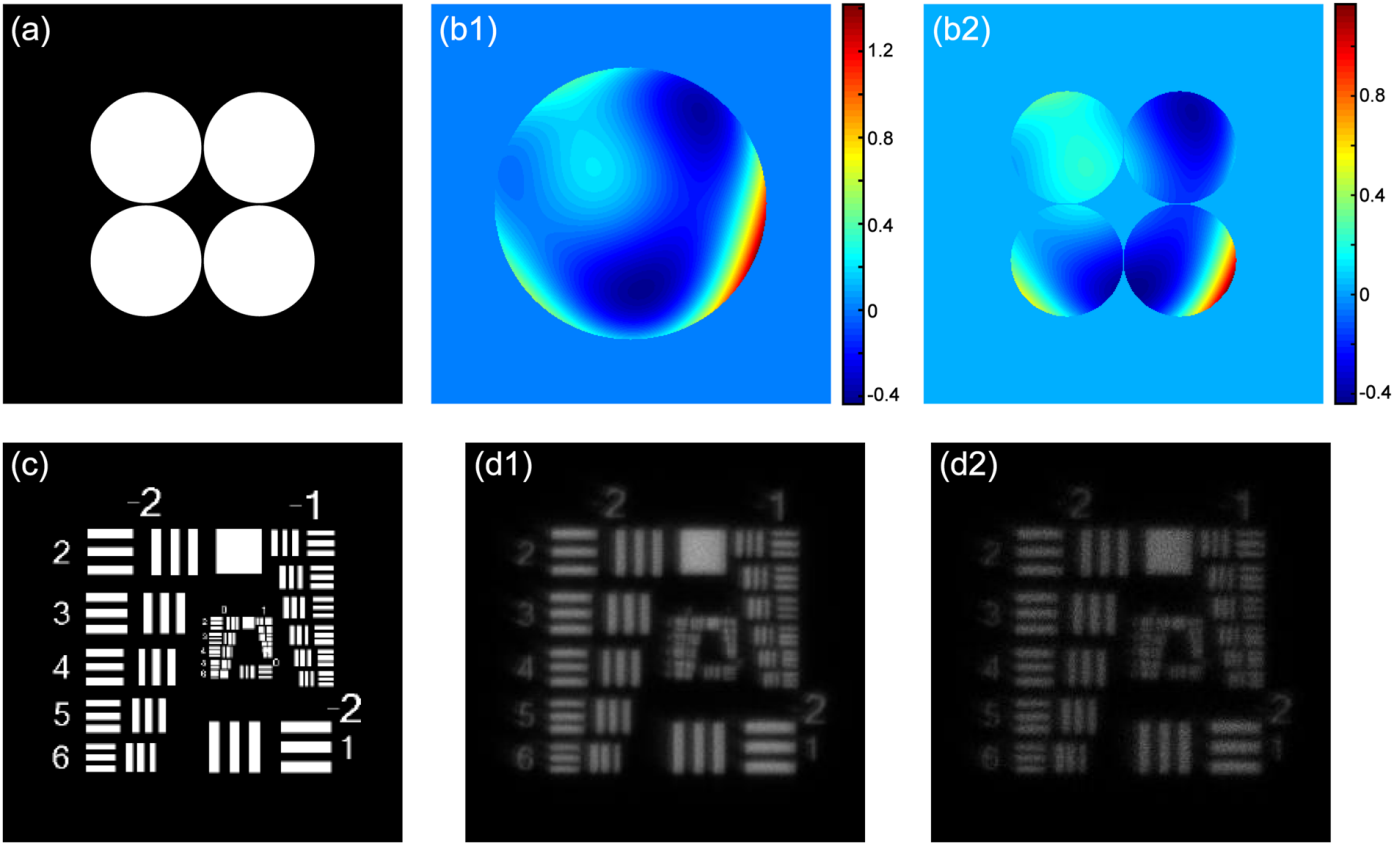
Simulation setup and simulated images. (**a**) The configuration of the simulated four aperture system with each sub-aperture adjacent to each other. (**b1**) The loaded global aberration generated by the first 11 Zernike polynomials (piston, x and y tilt excluded) and (**b2**) the segmented sub-aperture aberrations. (**c**) A USAF 1951 resolution test chart used here as the ideal object. The four sub-aperture synthetic images corresponding to PSNR values of (**d1**) 32.28 and (**d2**) 25.58 dB, respectively, which are distorted by the loaded aberrations. Their Co values are 0.8391 and 0.8271, respectively.

Simulations are conducted with monochromatic light of 632 nm to test the performance of the proposed ISMD. In the simulations, a sparse aperture system is built with four sub-apertures of diameters of 10 mm close to each other, shown as Fig. 1(a). The focal length is 500 mm and the pixel size of the CCD is 3.45 μm. A global wavefront of the imaging beam is generated by using the first 11 Zernike polynomials (piston, x and y tilt excluded) shown in Fig. 1(b1), of which the corresponding parts segmented by each sub-pupil are used to denote the sub-aperture aberrations, as shown in Fig. 1(b2). A USAF 1951 resolution test chart is used here as the faint object of our interest. We note that the faint object is simulated here by considering important photon noise. Then two groups of snapshot diversity images corrupted by the aberrations and noises are captured, corresponding to two different low photon levels. In each group, within a short time, 5 spatial modulations are completed, which are turning on all sub-apertures and turning off each one with the others open, thus generating a total of 5 frames of images. The raw images synthesized by 4 sub-apertures are shown as examples in Fig. 1(d1) and (d2), with PSNR values of 32.28 and 25.58 dB, respectively, which are of low quality due to the aberrations and photon noise. The corresponding Co values are 0.8391 and 0.8271, respectively. Other raw images are also at the similar photon levels of their corresponding synthetic images.

**Figure 2:**
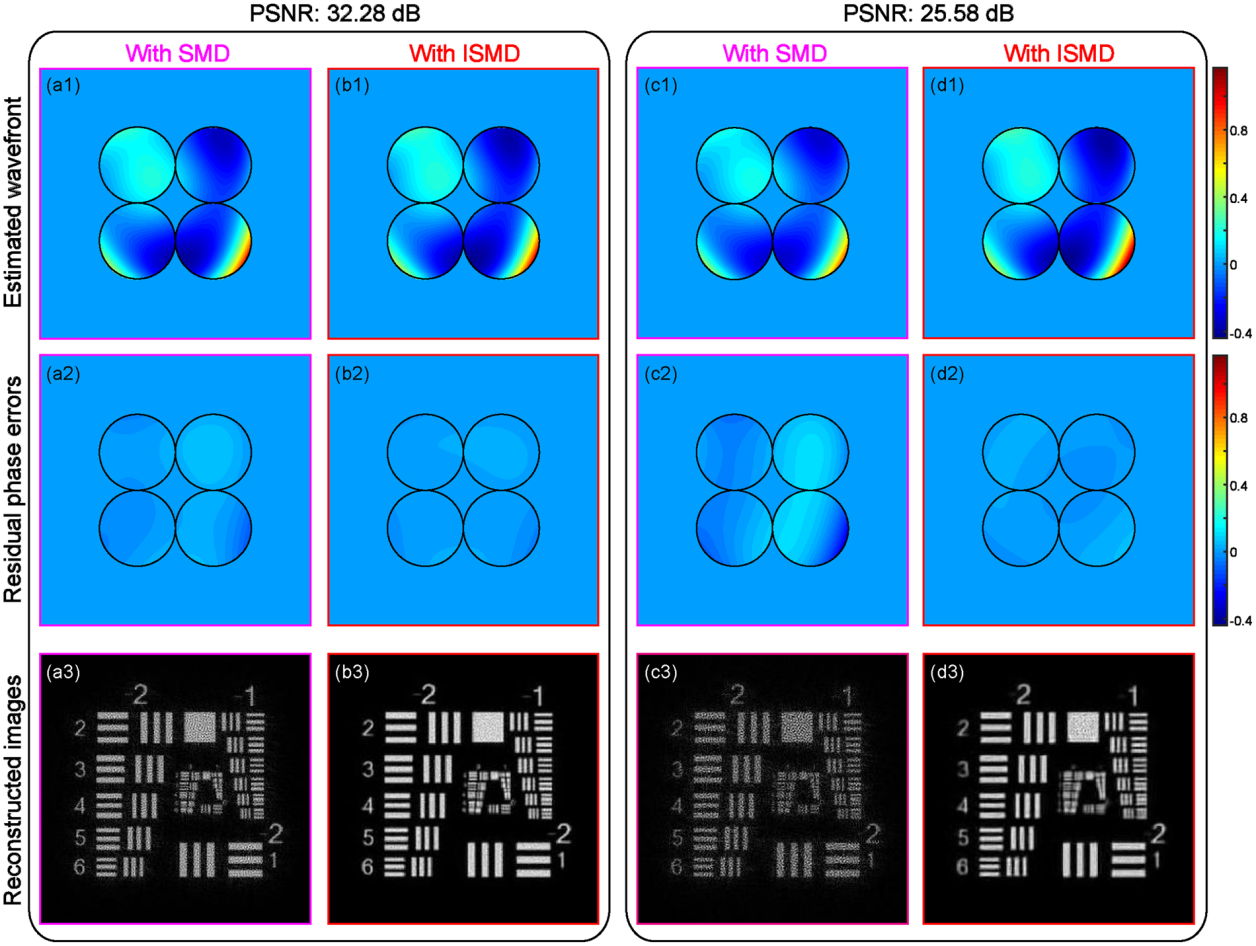
Comparison of simulation results with SMD and our proposed ISMD. The estimated wavefronts with (**a1**) SMD and (**b1**) ISMD for the case at photon level of PSNR of 32.28 dB; (**a2**)-(**b2**) the corresponding residual phase distributions, whose RMS errors are 0.0289 and 0.008 waves, respectively; (**a3**)-(**b3**) the corresponding reconstructed images, whose Co values are 0.9555 and 0.9889, respectively. The estimated wavefronts with (**c1**) SMD and (**d1**) ISMD for the case at photon level of PSNR of 25.58 dB; (**c2**)-(**d2**) the corresponding residual phase distributions, whose RMS errors are 0.0646 and 0.0137 waves, respectively; (**c3**)-(**d3**) the corresponding reconstructed images, whose Co values are 0.8645 and 0.9771, respectively.

Then based on the diversity images, both SMD and ISMD are handled to sense the wavefront and restore images with optimization procedures using the stochastic parallel gradient descent (SPGD) algorithm^16^. Figure 2 presents the results, from which it can be seen that ISMD generates superior reconstructions at both two photon levels. The estimated wavefronts from SMD at the PSNR levels of 32.28 and 25.58 dB are shown as Fig. 2(a1) and (c1), with corresponding residual phase errors presented in Fig. 2(a2) and (c2), whose RMS errors are 0.0289 and 0.0646 waves, respectively. The accuracy of the wavefront reconstructions are increased by our proposed ISMD. Figure 2(b1) and (d1) present its estimated wavefronts, and Fig. 2(b2) and (d2) show their corresponding residual phase distributions with RMS errors decreased to 0.008 and 0.0137 waves. It is also obvious to recognize that the two reconstructed images using ISMD from corresponding groups of noisy data sets present enhanced sharpness and more clear details, as shown in Fig. 2(b3) and (d3), with respect to those using SMD shown as Fig. 2(a3) and (c3). The Co values of the two recovered images with SMD are 0.9555 and 0.8645, while those with ISMD are increased to 0.9889 and 0.9771. From this set of simulations, it is shown that ISMD can exhibit better performance on wavefront sensing and image restoration than SMD when the sparse aperture system is observing a faint object.

### Experimental results

**Figure 3:**
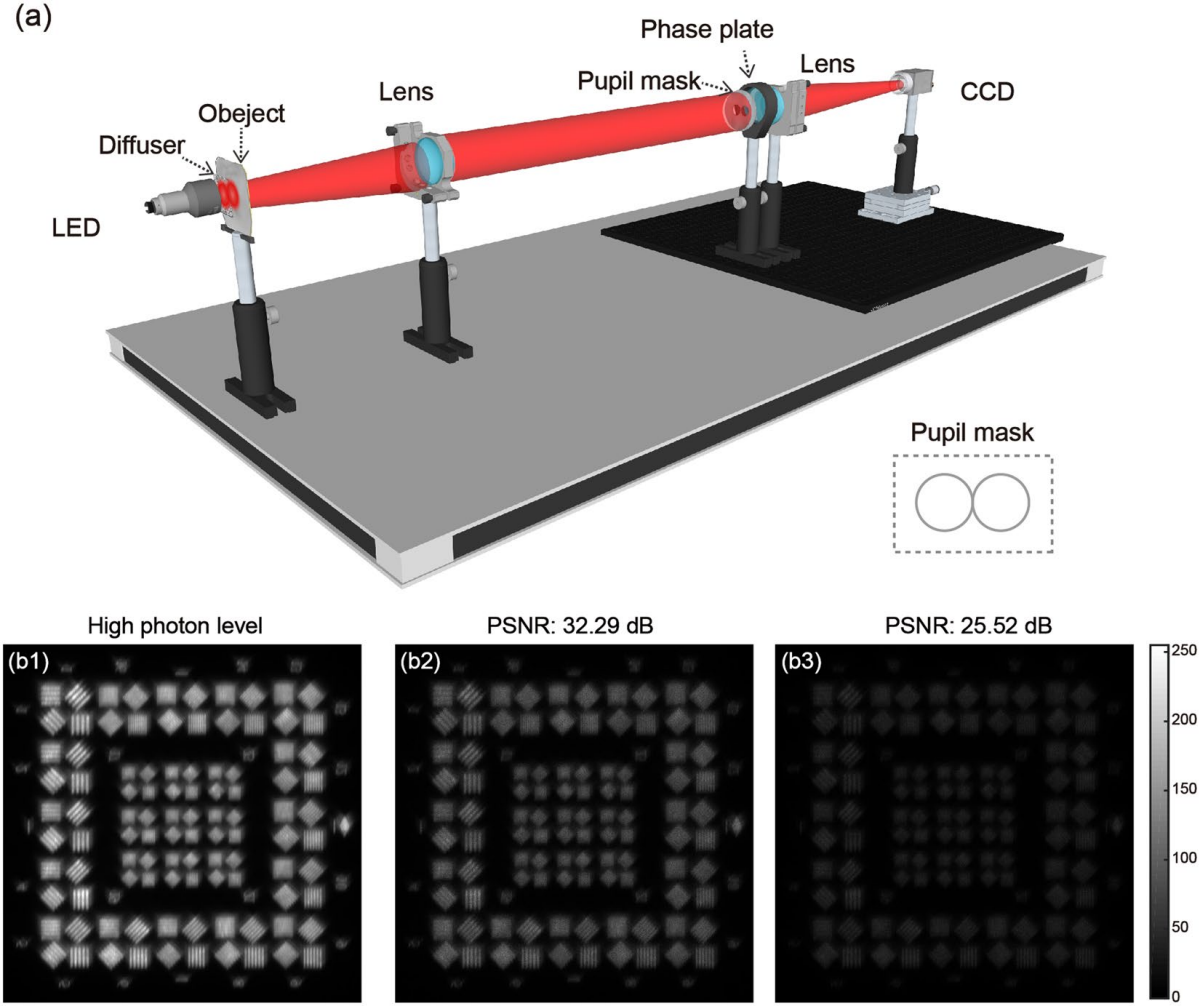
Experimental setup and raw images. (**a**) The configuration of the concept-demonstration experiment. A 630 nm LED is used to control the experimental photon levels. A binocular telescope is experimentally simulated by using a pupil-mask against an imaging lens, whose sub-aperture aberrations are characterized by a phase screen. The two sub-aperture synthetic images corresponding to (**b1**) a high photon level, and two low photon levels of PSNR of (**b2**) 32.29 and (**b3**) 25.52 dB, respectively.

The superior behavior of ISMD is also shown experimentally. We built an experimental setup, shown schematically as Fig. 3(a). A 630 nm LED source, whose intensity is adjustable, is used to illuminate a resolution test chart. We simulate an infinite condition by positioning the test chart on the focal plane of a lens. There is a diffuser utilized to uniform the illumination between the source and the target. It is easy to create faint objects by decreasing the LED energy in a dark condition. An imaging lens combined with a two-pupil mask is used to simulate a binocular telescope. The two sub-apertures are adjacent to each other. The diameter of each sub-aperture is 14 mm and the focal length of the imaging lens is 500 mm. Besides, we use a binary optics phase plate between the diaphragm and lens to characterize the sub-aperture aberrations. Specifically, the phase plate is a glass plate with varying thickness and its diameter is about 28 mm. The image is synthesized on the CCD camera (Point Grey GS3-U3-50S5M) with the pixel size of 3.45 μm.

Based on the setup, three sets of raw images at different photon levels are captured by the same spatial modulation. Specifically, one data set consists of one two-aperture and other two single-aperture images. We note that since single-aperture images generally have less photons than the synthetic ones, in the experiment the exposure time for each modulation of one dataset capturing is a little adjusted, just to keep the three raw images at the similar photon levels. A high photon level is achieved by maximizing the LED power and the corresponding images are recorded as the reference noiseless dataset, of which the two-aperture synthetic image is shown as Fig. 3(b1). Then by reducing the intensity of the LED, two sets of images of the relatively faint target at different photon levels are acquired, in which the two-aperture images are presented as Fig. 3(b2) and (b3), with PSNR values of 32.29 dB and 25.52 dB respectively, which are calculated approximately by taking the high photon synthetic image as the noiseless signals.

**Figure 4:**
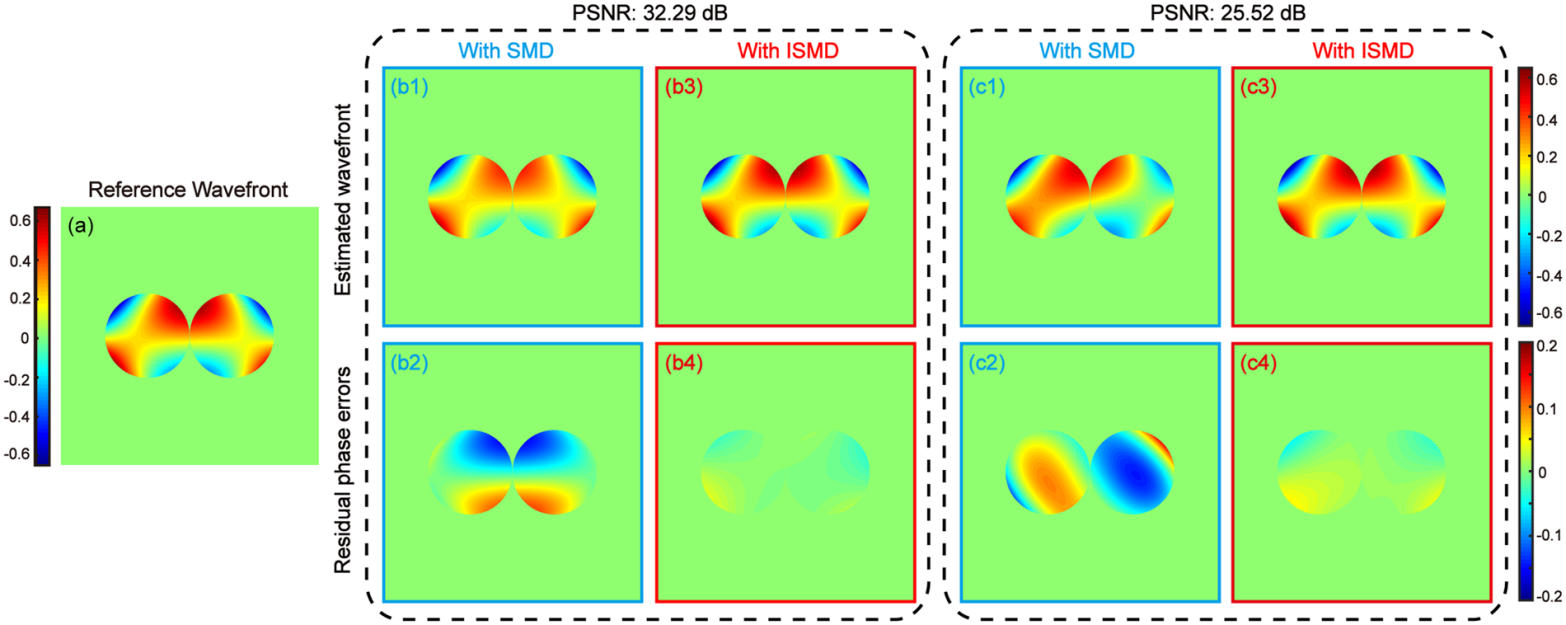
Comparison of experimental results of wavefront sensing with SMD and our proposed ISMD. (**a**) The reference wavefront reconstructed by using the high photon dataset. The reconstructed wavefronts with (**b1**) SMD and (**b3**) ISMD using the low photon dataset with PSNR of 32.29 dB; (**b2**) and (**b4**) the corresponding phase residual errors with respect to the reference wavefront, whose RMS errors are 0.0654 and 0.0106 waves, respectively. The reconstructed wavefronts with (**c1**) SMD and (**c3**) ISMD using the low photon dataset with PSNR of 25.52 dB; (**c2**) and (**c4**) the corresponding phase residual errors with respect to the reference wavefront, whose RMS errors are 0.0773 and 0.0186 waves, respectively.

It is necessary to test the accuracy of wavefront sensing, a crucial factor determining the quality of post-processing results. Here we use a clever repeatability RMS error to evaluate the degree of accuracy of wavefront reconstruction, which is introduced and also used in previous literature^11,17^. A reference wavefront is obtained by using SMD with the high photon dataset, as shown in Fig. 4(a). Then SMD and ISMD are both performed to process the other two datasets at different lower photon levels. The reconstructed wavefronts using SMD for different low PSNR levels of 32.29 and 25.52 dB are presented in Fig. 4(b1) and (c1), respectively, and their corresponding residual phase errors, which are defined as the differences between the estimated wavefronts and reference one in Fig. 4(a), are shown as Fig. 4(b2) and (c2) with RMS errors of 0.0654 and 0.0773 waves, respectively. Our proposed ISMD shows superior results. Figure 4(b3) and (c3) show the estimated wavefronts using ISMD for the different noise levels with their residual phase errors presented as Fig. 4(b4) and (c4). The corresponding RMS errors are decreased to 0.0106 and 0.0186 waves. From this set of experimental results, it is indicated that we can acquire more accurate wavefront reconstruction by ISMD than that by SMD when imaging fainter objects.

**Figure 5:**
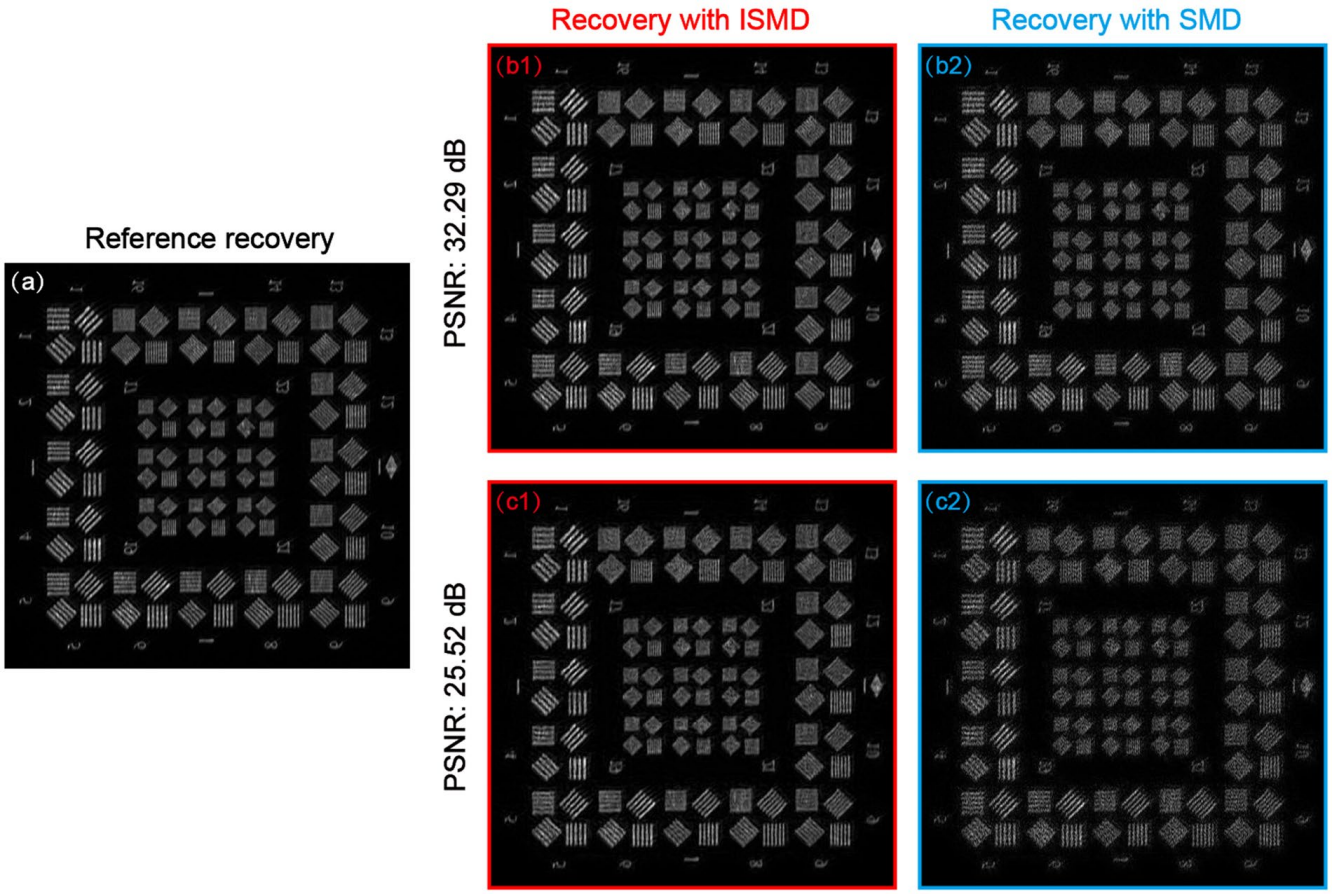
Comparison of experimental results of image restoration with SMD and our proposed ISMD. (**a**) The reference recovered image using the high photon dataset. The reconstructed images with (**b1**) ISMD and (**b2**) SMD using the low photon dataset with PSNR of 32.29 dB. The corresponding Co values are 0.9841 and 0.9027, respectively. The reconstructed images with (**c1**) ISMD and (**c2**) SMD using the low photon dataset with PSNR of 25.52 dB. The corresponding Co values are 0.9723 and 0.8368, respectively.

By means of more precise wavefront estimates, post-processed images of higher quality can be obtained. The results of image restoration are shown in Fig. 5. A high-resolution image presented as Fig. 5(a) is recovered as the reference by SMD using the high photon dataset. However, for low photon levels of PSNR of 32.29 and 25.52 dB, recovered images with SMD, shown as Fig. 5(b2) and (c2) respectively, exhibit unsatisfactory destruction grains and blurring with respect to the reference one in Fig. 5(a). It is an unsurprising result, since the raw images of the fainter objects suffer from serious photon noise that decreases the accuracy of wavefront sensing and degrades the images themselves. Luckily, the problem can be circumvented with our proposed ISMD. These raw images of fainter objects can be preprocessed to those matching higher signal-to-noise ratios, just like images of bright objects, with a state-of-the-art denoising technique termed the blocking-matching and 3D filtering algorithm. Based on the preprocessed datasets, we can get satisfactory reconstructed images shown as Fig. 5(b1) and (c1) corresponding to photon levels of PSNR of 32.29 and 25.52 dB, respectively. It is easy to recognize that compared with the image recoveries using SMD the ones using ISMD present enhanced sharpness, achieving the similar standard of quality to the reference recovery. We also calculate the Co values by taking Fig. 5(a) as the reference to quantify the experimental results. Corresponding to PSNR levels of 32.29 and 25.52 dB, the Co values of the two recovered images with SMD are 0.9027 and 0.8368, respectively, while those with ISMD are increased to 0.9841 and 0.9723. From the comparison, the superior image reconstruction using ISMD is demonstrated experimentally.

## Discussion

We have performed the simulations and experiments under similar conditions. The experimental results match the simulation results well. With the resolution test chart used as the object in both simulations and experiments, we can intuitively compare the results by observing the grains on the resolution bars. In experiments, the reconstructed images using ISMD present improved sharpness with fewer grains while those using SMD suffer from massive grains, especially at the lower photon levels, which coincides with the simulations. Also, the quantitative relationships in simulations and experiments also exhibit a good agreement. Specifically, at the two photon noise levels, the RMS values for ISMD in both simulations and experiments are smaller than those for SMD while the Co values for ISMD are bigger than those for SMD, verifying ISMD’s more accurate wavefront reconstruction and better image restoration.

**Figure 6:**
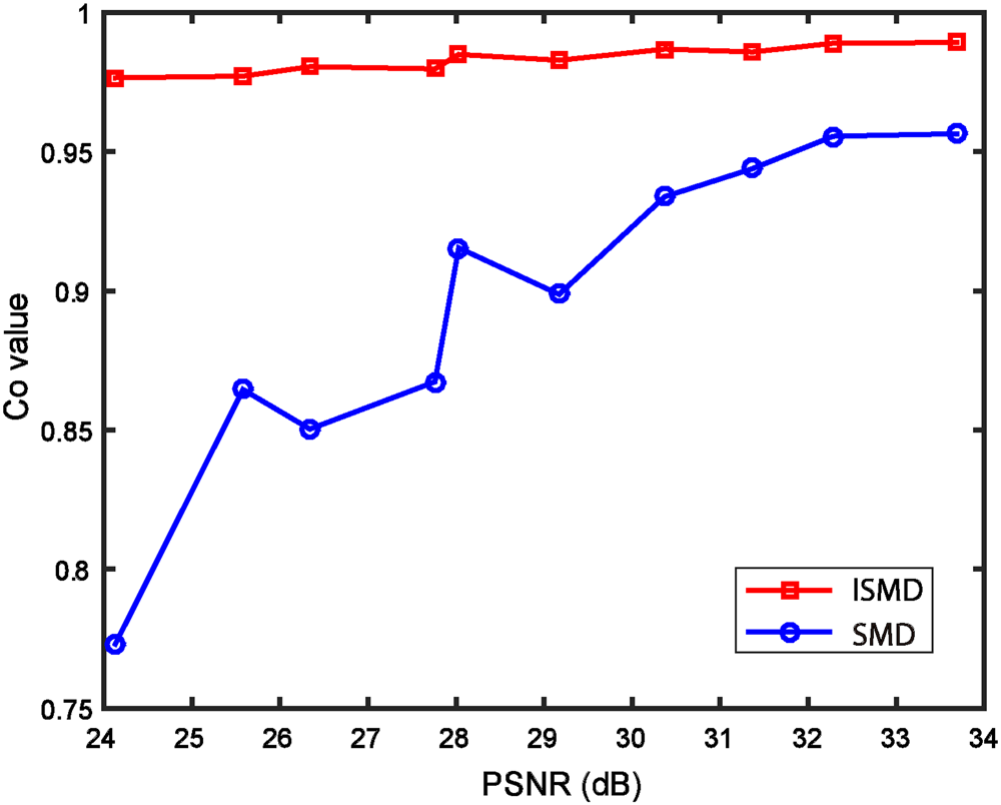
The evolution of the Co values with respect to the PSNR levels for both ISMD and SMD.

Furthermore, to explore the performance of ISMD for more photon levels, we investigate the Co variety associated with different PSNR values. After performing multiple repeatability simulations, we obtain the evolution of the Co values with respect to the different noise levels, shown as Fig. 6. From the curves, two points can be concluded to show the superiority of our proposed ISMD. On the one hand, the red line is always above the blue one within the testing noise range, implying that ISMD outputs superior images at all the test photon levels. On the other hand, the red line almost remains stable across different noise levels, indicating ISMD is robust against photon noises, while the blue one shows an obvious slope, indicating SMD is sensitive to photon noises and could only work at high photon levels.

It has been shown that ISMD proposed in this paper can exhibit better reconstructions than SMD when imaging a fainter objects. Actually, it is the ISMD’s capability of overcoming photon noise that has been demonstrated here, while the environment noises, such as CCD read noise and sky background, need to be taken into account in our future study. What’s more, it is also uncertain if ISMD could influence the photometric accuracy, which is also an interesting issue and needs our further investigation.

## Conclusion

Spatial modulation diversity is a newly developed post-processing technique for the future sparse aperture systems. Diversity images are generated by controlling the status of the transmittance of sub-apertures, which are required to be captured within a short time. When the objects are faint, the corresponding images will suffer from serious photon noises, degrading the accuracy of wavefront sensing and the quality of the recovered images. To address this problem, we propose and demonstrate an improved SMD with a denoising preprocessing procedure using the BM3D algorithm in this paper. The noisy raw images of the faint object are first preprocessed to those having high signal-to-noise ratios by BM3D algorithm, a state-of-the-art denoising technique. Then the previous SMD is applied to process the denoised raw images, extracting wavefronts and restoring images.

The superior performance of the proposed ISMD is verified by both simulations and experiments. The experiment and simulation results are in good agreement. In simulations, the RMS values of residual phase errors are decreased from 0.0289 and 0.0646 waves for SMD to 0.008 and 0.0137 waves for ISMD, at two low photon levels of PSNR of 32.28 and 25.58 dB, respectively. The Co values of the corresponding recovered images with SMD is 0.9555 and 0.8645, while those with ISMD are increased to 0.9889 and 0.9771. In the experiment, for the noise levels of 32.29 and 25.52 dB, smaller repeatability RMS errors indicate better results of wavefront sensing with ISMD than those with SMD. The quality of recovered images at the corresponding two low photon levels using ISMD outperforms that using SMD, almost matching the standard of the recovery at the high photon level. The Co values of recoveries using ISMD are 0.9841 and 0.9723 at the noise levels of 32.29 and 25.52 dB, respectively, while those using SMD are only 0.9027 and 0.8386. With the capability against photon noise much improved, the reported ISMD may find wide applications in the astronomical observations of faint objects of the future segmented mirrors or telescope arrays.

## Method

**Figure 7:**
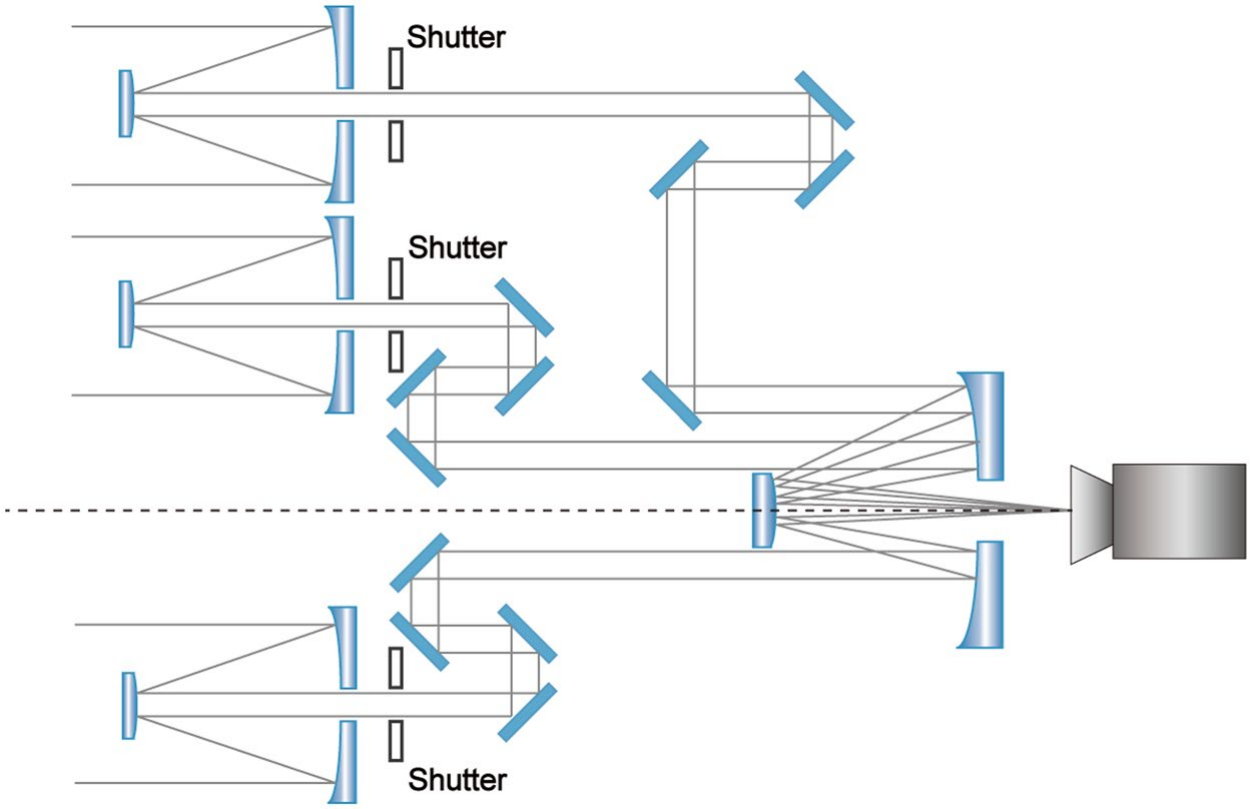
An example of the sparse aperture system, of which each sub-aperture is equipped with a shutter.

Spatial diversity modulation is a newly developed post-processing technique for the future telescope arrays or segmented mirrors, sharing its roots with phase diversity. A sparse aperture system with each sub-aperture equipped with a shutter is shown as Fig. 7. Based on such a scheme, SMD generates diversity images by controlling the transmittance of individual sub-aperture, respectively, instead of by means of focus adjustments. The on-off control can be implemented by spatial light modulators or digital micromirror devices in the future applications, which can help take multi-snapshots within a short time when the object of interest is in the isoplanatic patch. The aberrations of the multi-aperture system can be extracted from the diversity images with an optimization operation using stochastic parallel gradient descent (SPGD) algorithm, and then a high-quality image can be obtained with deconvolution.

The raw diversity images captured by CCD detector can be denoted as

4 $${d}_{k}(x)=o(x)\otimes {h}_{k}(x),$$

where ${d}_{k}(x)$ is the detected image, the subscript *k* represents the *k*th spatial modulation, $o(x)$ is the ideal intensity, ${h}_{k}(x)$ is the point spread function (PSF) of the system under the *k*th modulation, and $\bigotimes$ denotes a convolution. According to incoherent imaging theory^18^, the PSF ${h}_{k}(x)$ can be modeled as follows

5 $${h}_{k}(x)={|{ {\mathcal F} }^{-1}(\sum _{n=1}^{N}{S}_{n,k}(u)\times {p}_{n}(u)\times \exp (i\sum _{m=1}^{M}{\alpha }_{m}{Z}_{m}(u)))|}^{2},$$

where *p*_*n*(_ *u*) is the binary pupil function of the *n*th sub-aperture, ${S}_{n,k}(u)$ stands for the status of the transmittance of the *n*th sub-aperture in the case of the *k*th modulation, ${Z}_{m}(u)$ is the *m*th order Zernike polynomial^19^, ${\alpha }_{m}$ are the Zernike expansion coefficients used as the control parameters, *M* is the total order of Zernike polynomials, and *N* is the number of all the sub-apertures. Specifically, in this paper, the spatial modulation is realized by turning off each sub-aperture alternatively, so ${S}_{n,k}(u)$ can be expressed as follows

6 $${S}_{n,k}(u)=\{\begin{array}{cc}0 & n=k\\ 1 & n\ne k\end{array},$$

where *n* = 1, 2, 3, …, *N* and *k* = 0, 1, 2, …, *N*. *k* = 0 means opening all the sub-apertures.

In this way, the PSF is modeled as a function with respect to Zernike coefficients that are ready to be optimized. Based on maximum likelihood framework, an optimization metric in Fourier domain can be expressed as

7 $$E(f,\alpha )=\sum _{f}\sum _{k=1}^{K}{|{D}_{k}(f)|}^{2}-\sum _{f}\frac{{|{D}_{k}(f){H}_{k}^{\ast }(f)|}^{2}}{{\sum }_{k=1}^{K}{|{H}_{k}^{\ast }(f)|}^{2}},$$

where *f* is a 2D vector in Fourier domain, ${D}_{k}(f)$ and ${H}_{k}(f)$ are 2D Fourier transforms of ${d}_{k}(x)$ and ${h}_{k}(x)$, and * denotes complex conjugation operator. SPGD algorithm, which could achieve the maximum convergence speed when optimizing Zernike polynomials^19^, is used here to minimize the metric above. How the algorithm works can be referred in detail from our previous paper^7^. After the optimization procedure, we can estimate the aberrations of the sparse aperture system by summing up the Zernike polynomials with optimized coefficients. Finally, the object can be reconstructed with the following equation.

8 $$O(f)=\frac{{\sum }_{k=1}^{K}{D}_{k}(f){H}_{k}^{\ast }(f)}{{\sum }_{k=1}^{K}{|{H}_{k}(f)|}^{2}},$$

The diversity images of SMD are required to be captured within a short time when the object is in the isoplanatic patch. Thus when fainter objects are observed, the raw images will suffer from serious Poisson noise due to the photon detection. The randomly distributed noises would submerge some intensity details and break down the diversity relationships of raw images, thus decreasing the accuracy of wavefront sensing. Besides the degradation of estimated wavefront, the final reconstructed image is also corrupted by the noise itself. This problem limits the SMD to further applications in astronomical observations of future sparse aperture systems.

In this paper, we propose an improved SMD to address the issue, which involves a denoising preprocessing step with respect to the previous technique. The blocking-matching and 3D filtering algorithm^14,15^, a state-of-the-art denoising technique, is first used to preprocess the raw images of fainter objects suffering serious noises. In the beginning of the denoising procedure, similar 2D image blocks are grouped into 3D data arrays. Then the collaborative transform-domain shrinkage is used to deal with these 3D groups following three successive steps: 3D linear transformation of a group, shrinkage of the transform coefficients for noise reduction, and inverse 3D linear transformation. Finally, an estimate of the noiseless image is acquired by aggregating estimates of all the grouped blocks with a weighted average. As a result, raw noisy diversity images are transformed equivalently to those corresponding to bright objects with high signal-to-noise ratios. Based on these denoised images, traditional SMD can be then applied, and better wavefronts and images can be reconstructed. In this way, we reduce the degradation of the photon noise, and the proposed ISMD is suitable for observations of relatively faint objects of future telescope arrays or segmented mirrors.
